# Poliomyelitis in Bristol, 1950

**Published:** 1952-01

**Authors:** James Macrae

**Affiliations:** Resident Physician, Ham Green Hospital


### POLIOMYELITIS IN BRISTOL, 1950

BY

JAMES MACRAE, M.D., F.R.F.P.S., D.P.H.

*Resident Physician, Ham Green Hospital*

Successive reports* in this *Journal* on the progress of poliomyelitis in the Bristol area serve to co-relate the disease with other infections but reduce what can be usefully said that is new about this much publicised disease.

Therefore, it seems best to place before you the bare bones of the incidence during 1950, as reflected in admissions recorded at Ham Green Hospital, and thereafter comment only on what appears to be of practical interest.

### INCIDENCE. AGE. SEVERITY.

† 1950 Ham Green Hospital, Bristol, 316 cases = 60·2 per 100,000

| Age | 0—1 | | 1—4 | | 5—14 | | 15—24 | | 25+ | | Totals | | Grand total |
|---|---|---|---|---|---|---|---|---|---|---|---|---|---|
| Sex | M | F | M | F | M | F | M | F | M | F | M | F | |
| Non-paralytic | — | 2 | — | 3 | 5 | 3 | 3 | 1 | 4 | 2 | 12 | 11 | 23 |
| Slight | 3 | 1 | 9 | 6 | 12 | 7 | 3 | 2 | 1 | 3 | 28 | 19 | 47 |
| Moderate | 5 | 8 | 18 | 23 | 21 | 19 | 8 | 7 | 6 | 10 | 58 | 67 | 125 |
| Severe | 9 (4) | 6 (2) | 21 (1) | 16 (2) | 22 (6) | 16 (6) | 7 (2) | 2 | 17 (6) | 5 (3) | 76 (19) | 45 (13) | 121 (32) |
| Totals | 17 | 17 | 48 | 48 | 60 | 45 | 21 | 12 | 28 | 20 | 174 | 142 | 316 |
| Grand total | 34 (6) | | 96 (3) | | 105 (12) | | 33 (2) | | 48 (9) | | 316 (32) | | |

Deaths in parentheses. Extremes of age: 4 weeks to 52 years.

† Approximate national rate per 100,000 for 1950 = 17.

These figures indicate an incidence of infection never previously experienced in Bristol and more severe than has been so far reported in any comparable area of Britain.

The disease occurred between July and December with its peak late in September when as many as twenty-five cases were admitted each week. Eighty-

* Macrae, J., *Bristol Medico-Chirurgical Journal* 1947, LXIV, p. 101; 1950, LXVII, p. 43.

eight patients were sent to hospital as suffering from poliomyelitis but were later proved to be:

| | |
|---|---|
| Tonsillitis .. .. .. .. | 35 cases. |
| Pneumonia .. .. .. .. | 10 cases. |
| Other central nervous diseases .. | 7 cases. |
| Otitis media .. .. .. .. | 4 cases. |
| Septicaemia .. .. .. .. | 4 cases. |

and thereafter odd cases of sinusitis, cervical adenitis, measles, scarlatina, dysentery, constipation, pyelitis, arthritis, fibrositis, acute rheumatism, asthma, typhoid fever, diabetic coma, Weil's Disease, and pregnancy. Thirty-nine cases of poliomyelitis were admitted under some other diagnosis, notably meningitis.

The large total number (316) and the high proportion of severely ill patients laid a strain on the hospital resources. For a time nine mechanical respirators were in simultaneous use and over a hundred patients were under treatment. During September and October a hundred and ninety-one patients were admitted. Without nursing help from other hospitals we could have continued treatment of poliomyelitis only at the expense of other infections. As a result of this experience several aspects of poliomyelitis have been clarified.

### FAMILY INCIDENCE

The members of a family appear to become infected with poliomyelitis virus almost simultaneously as if from a common source. One who develops paralysis is the clinical case among the infected group, rather than the source of infection for the others. Several paralytic cases in one family are not uncommon, and we have had as many as four.

### BULBAR-ENCEPHALITIC TYPES

In our 1950 series no less than 158 cases showed bulbar or encephalitic signs. These included all the most severe cases and many relatively mild ones, depending largely on the pathological accident of which nuclei were most heavily attacked. Destruction of vital nuclei can cause death very quickly. When sudden death occurs in a patient who has had symptoms of encephalitis or of severe toxaemia the diagnosis of poliomyelitis should be excluded only after histological examination of the brain and spinal cord.

Cases requiring mechanical respiration are practically all in this group, as indeed were all the fifty-three patients we had in respirators during 1950. Lesions affecting respiration usually affect other vital centres, and so we found that twenty-seven of our respirator patients died.

Patients with severe pharyngeal paralysis, either with or without paralysis of respiration, are easily the most difficult to deal with but they are worth great effort in trying to prevent them drowning in their own secretion. Gravity drainage for these cases seems simplest and best.

The bright side of the bulbar type of lesion shows if the patient can be carried through his acute phase, for he will often recover full function. Patients appear to recover completely from the encephalitis of poliomyelitis.

### FATIGUE

The work of Russell and Horstmann has made plain the bad influence of exercise on the incidence of paralysis, and McCloskey drew attention to the possible evil influence of inoculation. Among our cases we found plenty of evidence to support Dr. Ritchie Russell's conclusions, and the only form of treatment of the active virus stage of the disease remains his suggestion that all possible poliomyelitis victims should rest absolutely from the first sign or symptom. Inoculations did not figure much in the histories of our cases. At the same time, it is very reasonable to suppose that the extra fatigue of exercise, trauma, or inoculation, might well be the deciding factor between a live or a dead nerve cell at the time when the motor neurones are entertaining myriads of virus particles.

### NON-PARALYTIC TYPES

In the absence of virus culture the non-paralytic case must always remain clinically doubtful. It is at best a negative diagnosis, and while it is undoubtedly a very prevalent clinical entity, the diagnosis should not be lightly made. As one example, one wonders how many non-paralytic cases are due to Coxsackie virus.

### TAIL PIECE—1951

Despite the portents of another severe summer incidence of poliomyelitis, Ham Green Hospital has up to now (October 1951) admitted forty-seven cases with two deaths. The cases have been much milder than in 1950 and only one has required the mechanical respirator. Something has presumably happened in the immunology of the disease but no doubt there is still a likelihood of severe outbreaks in Bristol in the future.

### REFERENCES

Russell, W. R., *Brit. Med. J.* 1947, ii, 1023.
Horstmann, D. M., *J. Amer. Med. Ass.*, 1950, **142**, 236.
McCloskey, B. P., *Lancet*, 1950, i, 659.

